# Distinct Host Tropism Protein Signatures to Identify Possible Zoonotic Influenza A Viruses

**DOI:** 10.1371/journal.pone.0150173

**Published:** 2016-02-25

**Authors:** Christine L. P. Eng, Joo Chuan Tong, Tin Wee Tan

**Affiliations:** 1 Department of Biochemistry, Yong Loo Lin School of Medicine, National University of Singapore, Singapore, Singapore; 2 Institute of High Performance Computing, Singapore, Singapore; Centre of Influenza Research, The University of Hong Kong, HONG KONG

## Abstract

Zoonotic influenza A viruses constantly pose a health threat to humans as novel strains occasionally emerge from the avian population to cause human infections. Many past epidemic as well as pandemic strains have originated from avian species. While most viruses are restricted to their primary hosts, zoonotic strains can sometimes arise from mutations or reassortment, leading them to acquire the capability to escape host species barrier and successfully infect a new host. Phylogenetic analyses and genetic markers are useful in tracing the origins of zoonotic infections, but there are still no effective means to identify high risk strains prior to an outbreak. Here we show that distinct host tropism protein signatures can be used to identify possible zoonotic strains in avian species which have the potential to cause human infections. We have discovered that influenza A viruses can now be classified into avian, human, or zoonotic strains based on their host tropism protein signatures. Analysis of all influenza A viruses with complete proteome using the host tropism prediction system, based on machine learning classifications of avian and human viral proteins has uncovered distinct signatures of zoonotic strains as mosaics of avian and human viral proteins. This is in contrast with typical avian or human strains where they show mostly avian or human viral proteins in their signatures respectively. Moreover, we have found that zoonotic strains from the same influenza outbreaks carry similar host tropism protein signatures characteristic of a common ancestry. Our results demonstrate that the distinct host tropism protein signature in zoonotic strains may prove useful in influenza surveillance to rapidly identify potential high risk strains circulating in avian species, which may grant us the foresight in anticipating an impending influenza outbreak.

## Introduction

Influenza A viruses remain a public health threat with annual recurrence of seasonal influenza viruses in addition to sporadic avian influenza outbreaks in human population as well as rare, but formidable pandemic events. While most viruses are restricted to their primary hosts, zoonotic strains can sometimes arise through mutations or reassortment events, leading them to acquire the capability to escape host species barrier and successfully infect a new host [1]. The devastating consequences resulting from these zoonotic strains are evident from the highly pathogenic H5N1 outbreaks in Asia and Africa [2,3], as well as the H7N9 outbreak in China [4,5], which carried high rates of morbidity and mortality. Zoonotic strains are therefore a serious concern and it would be prudent to identify these strains prior to an outbreak for appropriate measures to be taken.

Current influenza surveillance focuses on surveillance, detection and response following influenza outbreak [6]. Virus strains are rapidly identified and characterized antigenically as well as genetically, with phylogenetic analyses performed to trace the origins of the infections [7]. Mutations on notable species-associated genetic markers are sometimes identified to further characterize the adaptation of the virus strain from avian to mammalian hosts [8,9]. However, most of these host switch events are unique and the genetic markers may not be applied to novel strains of different subtypes. Furthermore, efficient transmission and adaptation of avian viruses to humans is a complex process involving many factors. Even with the intensive research on influenza viruses, we are still no closer to predicting zoonotic strains having the ability to cause outbreaks in human population.

As opposed to commonly used approaches focusing on molecular adaptations of interspecies transmission, we adopted a systemic approach which considers the contribution of 11 influenza virus proteins to look for host tropism protein signatures prevalent in avian, human, and zoonotic strains. In this study, we utilized an influenza host tropism prediction system to obtain independent host tropism predictions of 11 influenza virus proteins (HA, M1, M2, NA, NP, NS1, NS2, PA, PB1, PB1-F2, and PB2) [10], the results of which were concatenated to provide an overview of the underlying host tropism protein signatures for influenza virus strains. Host tropism protein signature is herein defined as an influenza viral proteome profile of 11 independent avian or human protein predictions classified by the host tropism prediction system. We thus sought out to investigate the host tropism protein signatures of all influenza A virus strains, and to examine differences in the signatures of avian, human and zoonotic strains.

## Methods

### Influenza protein sequences

Protein sequences of influenza A virus strains were obtained from Influenza Research Database (http://www.fludb.org) [11]. The initial dataset consists of 331,748 protein sequences of HA, M1, M2, NA, NP, NS1, NS2, PA, PB1, PB1-F2, and PB2 from 61,559 strains. Virus strains with invalid protein sequences, incomplete proteome, and sequence discrepancy were next removed. Furthermore, we observed that prediction accuracies decrease for protein sequences that are of incomplete length (S2 Fig), justifying the exclusion of these sequences from further analyses. The full host tropism protein signature analysis thus involves 12,624 avian and human-isolated strains with 11 complete full-length protein sequences.

A crucial step in this study involved identifying confirmed zoonotic influenza virus strains isolated from human patients during influenza outbreaks. We have identified a total of 126 confirmed zoonotic strains as well as a further 346 avian strains isolated from the corresponding zoonotic outbreaks as suspected zoonotic strains (Table 1, S1 Dataset). These zoonotic strains were identified based on published literature on zoonotic or avian influenza outbreaks [4,12–32], United States Center for Disease Control and Prevention (CDC) reports [33–37], as well as World Health Organization (WHO) reports [38–43].

**Table 1:** Summary of confirmed and suspected zoonotic strains with complete proteome identified.

Year	Subtype	Country	Avian-isolated suspected zoonotic strains	Human-isolated confirmed zoonotic strains	Reference
1997	H9N2	Hong Kong	2		[24]
1998	H9N2	China	1		[24]
1999	H9N2	China	2		[24]
2003	H7N7	Netherlands	1		[20]
	H9N2	Hong Kong	2		[35]
2004	H5N1	Thailand	10		
		Vietnam	14		[35,39]
	H7N3	Canada		1	[18,35]
2005	H5N1	Cambodia		1	[39,44]
		China		1	[39,44]
		Indonesia	6	7	[26,39,44]
		Thailand	24		[39,44]
		Vietnam	46		[39,44]
2006	H5N1	Egypt	3	2	[39]
		Indonesia	9	46	[39,45]
		Iraq		1	[39,45]
		Thailand	7	1	[39]
		Turkey		3	[29,32,46]
		Vietnam	2		[39]
2007	H3N8	Laos	1		[39]
	H5N1	Indonesia	2	10	[39]
		Laos	16		[39]
		Nigeria	20		[39]
		Vietnam	51		[39]
2008	H3N8	Vietnam	1		[39]
	H5N1	Bangladesh		1	[39]
		China	1		[39]
		Egypt	18		[39]
		Vietnam	1		[39]
	H9N2	Hong Kong	9		[24]
	H11N9	Vietnam	2		[39]
2009	H5N1	Cambodia	1		[39]
		Egypt	6		[39]
	H9N2	Hong Kong	4		[47]
	H12N5	Vietnam	1		[39]
2010	H5N1	Cambodia	5	1	[39]
		China		1	[39]
		Egypt	25		[36,39]
2011	H5N1	Bangladesh	1	1	[38,39]
		Cambodia	6	4	[38,39]
		Egypt	13		[38,39]
2012	H5N1	Cambodia		1	[2]
	H7N3	Mexico		1	[37]
2013	H5N1	Cambodia	1	4	[2]
	H7N9	China	32	31	[40,41]
		Hong Kong		1	[40]
		Taiwan		2	[40]
	H10N8	China		4	[48]
2014	H7N9	China		1	[40,41]
		**Total**	**346**	**126**	

### Host tropism protein signature analysis

The host tropism protein signature analysis was performed using an influenza host tropism protein signature prediction system (http://fluleap.bic.nus.edu.sg) [10]. The host tropism prediction system consists of 11 individual protein prediction models (HA, M1, M2, NA, NP, NS1, NS2, PA, PB1, PB1-F2 and PB2) which independently predicts the host tropism of each protein given the protein sequences. Each protein prediction model was constructed using the machine learning algorithm random forest trained on datasets of avian and human protein sequences, which were transformed into machine learning feature vectors using amino acid compositions as well as physicochemical properties. Differences in the amino acid compositions and physicochemical properties between avian and human protein sequences allowed the prediction models to accurately distinguish between avian and human proteins with a minimum of 96.57% and up to 98.62% accuracy [10]. Results from the 11 individual avian or human protein predictions could therefore be combined for each influenza virus strain, characterizing its host tropism protein signature. We then analysed the signatures based on four groups of influenza viruses: typical avian and human strains circulating in avian and human hosts respectively, confirmed zoonotic strains isolated from human patients during zoonotic outbreaks and suspected zoonotic strains isolated from avian sources during zoonotic outbreaks.

We next performed hierarchical clustering on the host tropism protein signatures of suspected and confirmed zoonotic strains. The signatures comprising of avian and human predictions for each protein were represented with binary values and standardized for comparability. The clustering was performed using Ward’s minimum variance method with Euclidean distance metric [49]. Only avian-isolated suspected zoonotic strains with at least one human protein in their signatures were included.

## Results

**Fig 1:**
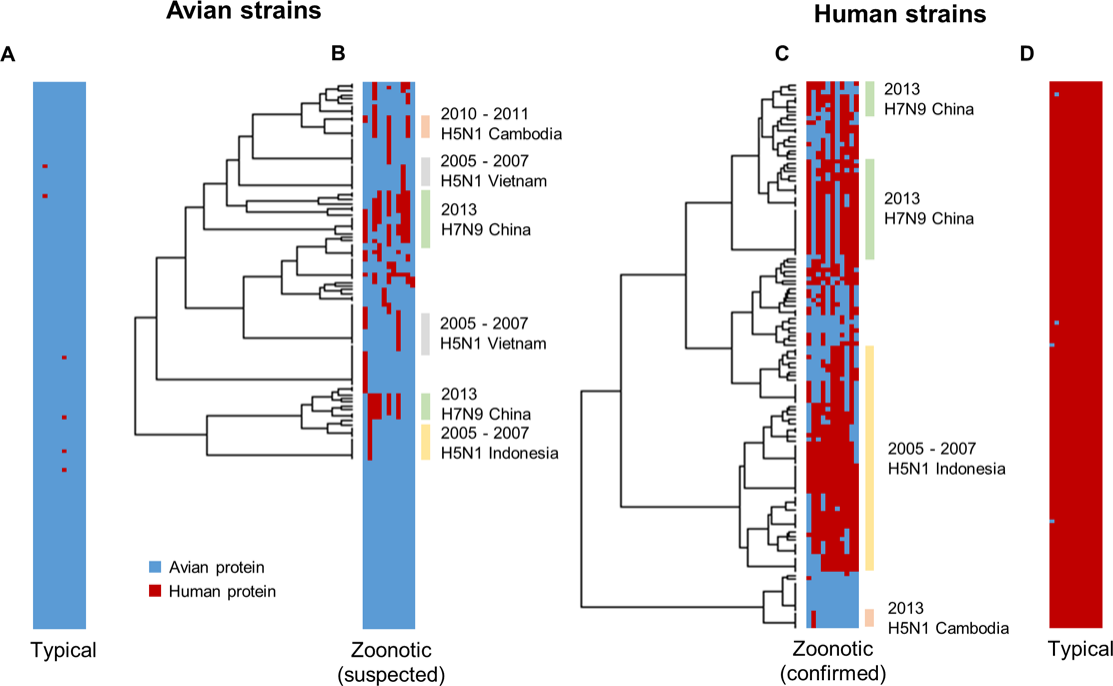
Host tropism protein signatures of avian, human and zoonotic strains. Each row in the bar represents a strain, with 11 columns depicting one of each protein prediction (HA, M1, M2, NA, NP, NS1, NS2, PA, PB1, PB1-F2, and PB2). Blue column indicates an avian protein prediction while red column indicates a human protein prediction. Only a total of 146 strains are shown in each bar to match the low number of samples in confirmed zoonotic strains, with the complete result landscape illustrated in S1 Fig. (A) Typical avian strains show almost exclusive avian predictions. (B) Suspected zoonotic strains isolated from avian species during zoonotic outbreaks show slight mosaic patterns of mixed avian and human predictions. (C) Confirmed zoonotic strains isolated from human patients during zoonotic outbreaks display prominent mosaic patterns. (D) Typical human strains with almost exclusive human predictions. Hierarchical clustering shows that zoonotic strains from the same outbreaks tend to have similar signatures, with the bars indicating strains from the zoonotic outbreaks.

**Fig 2:**
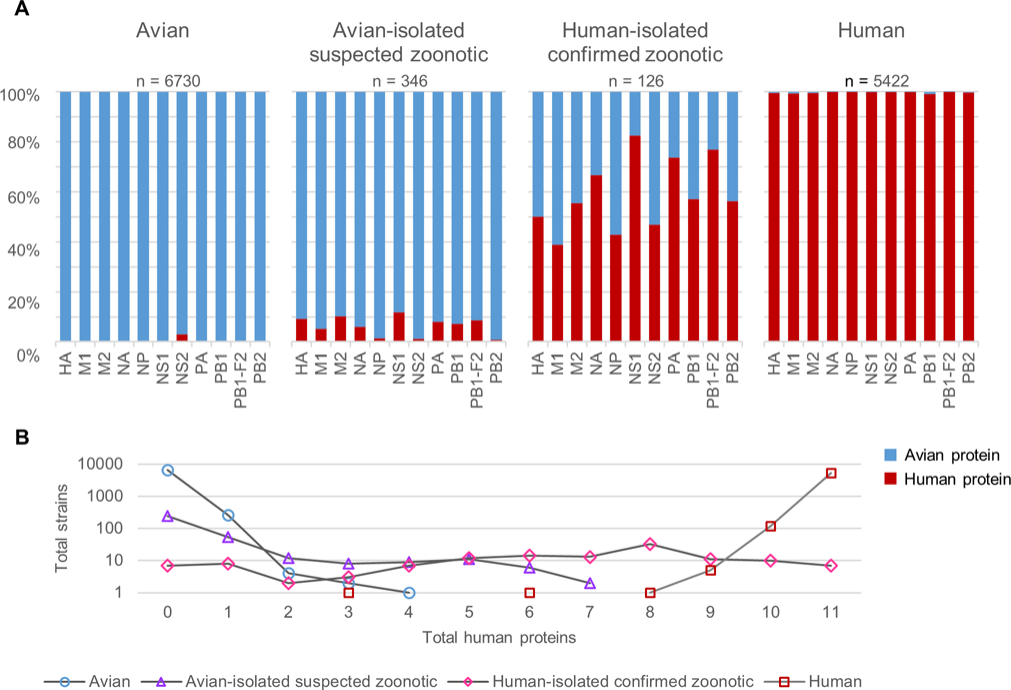
Percentage predictions and total number of human proteins for avian, human and zoonotic strains. (A) 100% stacked bars representing total avian and human predictions for each protein in each group. (B) Total number of human proteins in the host tropism protein signatures of each group. A logarithmic scale of base 10 was applied to total samples.

Analysis of the host tropism protein signatures show reveal indeed, there are distinct signatures between avian, human and zoonotic strains. Almost all avian strains display a unanimous signature of entirely avian proteins (Fig 1A). The percentage of avian to human predictions for each protein exceeds 99% except for NS2, where 3% of the predictions are human proteins (Fig 2A). Merely 3.86% of the avian strains have one human protein while the remaining 0.10% have at most two to four human proteins in their signatures (Fig 2B). These findings suggest that most circulating avian strains carry only avian proteins, thus rendering most of them incapable of crossing the host species barrier to freely infect a new secondary host.

Through accumulation of mutations or acquiring new gene segments from reassortment [50], avian strains may over time evolve to harbour human proteins in their proteome, as evident from the host tropism protein signatures of avian strains isolated during zoonotic outbreaks (Fig 1B). While more than half still carry the typical avian signature of 11 avian proteins and only 15.32% have one human protein in their signatures (Fig 2B), the remaining 13.87% were observed to have a combination of between two to seven human proteins (Fig 2B). Additionally, hierarchical clustering results of suspected zoonotic strains with at least one human protein reveal that strains isolated from the same outbreaks share similar host tropism protein signatures. These findings suggest that some of these avian strains could be the possible source of zoonotic infections.

We next speculate that as these avian strains evolve to acquire more human features in their proteins, they may eventually escape their primary host species barrier and successfully infect human hosts. This can be observed in the host tropism protein signatures of confirmed zoonotic strains isolated from human hosts during outbreaks (Fig 1C), where in contrast to typical avian and human strains, these zoonotic strains clearly display a mosaic of mixed avian and human proteins. Consistent with earlier clustering results of suspected zoonotic strains, strains isolated from the same zoonotic outbreaks were clustered in the same groups (Fig 1C). This therefore strongly indicates that zoonotic strains isolated from the same outbreak exhibit similar host tropism protein signatures.

The ratio of human to avian protein predictions increased tremendously for confirmed zoonotic strains as compared to suspected zoonotic strains, with as many as five proteins (M2, NA, NS1, PA, PB1-F2) having more than 50% human predictions (Fig 2A). Different human to avian protein prediction ratios signifies that some proteins may play more important roles than others in host switch events. It comes as no surprise that proteins with the least percentage of human predictions were M1 and NP (Fig 2A), which serve primarily structural roles within the virus [51]. Perhaps the more astonishing of which is that well-known protein determinants of host adaptation such as HA and PB2 were not among the top proteins with the most human predictions, but rather, NS1 and PB1-F2 with less characterized roles in interspecies transmission (Fig 2A).

HA has long been acknowledged as a crucial protein determinant in cross-species transmission, with the specificity for different types of sialic acid species determining binding to avian or human host receptors [52–54]. However, we observed here that only 50% of the confirmed zoonotic strains have human HA (Fig 2A), noting that it is not a requirement for a switch in receptor specificity for efficient transmission to humans, a sentiment also echoed by recent studies [1]. Similarly, the PB2 polymerase protein, especially at position 627 [9,55], has been identified as another critical determinant of host range, affecting efficient viral transcription and replication in host cells [56]. Yet only slightly more than half of the PB2 proteins in confirmed zoonotic strains were predicted as human (Fig 2A), with the other half retaining functions more similar to proteins in avian strains, the effect of which is probably compensated by other polymerase proteins.

Surprisingly, NS1 and PB1-F2 protein were predicted to be human in as much as 82.95% and 76.94% respectively of the confirmed zoonotic strains (Fig 2A), strongly supporting their major contributions in avian-to-human transmission. Despite that, both proteins are more widely known to be associated with virulence and pathogenicity [57,58], with their roles in host range restriction uncertain. Nevertheless, NS1 functions to inhibit host immune response and there are studies suggesting that efficiency in controlling interferon response can affect host range [59]. Based on the prevalence of human NS1 and PB1-F2 in confirmed zoonotic strains, our study highlights the importance of both proteins in interspecies transmission, even more so than previously thought. Future molecular studies are warranted to link the functions of both proteins with interspecies transmission.

Additionally, almost a third of confirmed zoonotic strains carry between five to ten human proteins in the signatures (Fig 2A), suggesting that an avian strain need not acquire all human proteins to be able to successfully infect humans. In fact, this further highlights the complexity of interspecies transmission, whereby it requires the contribution of more than a single protein to grant a virus the ability to escape host species barrier. We are therefore required to move on from relying heavily on single markers in determining whether an avian strain has zoonotic ability, to analysing the influenza virus strain from a systems perspective by looking at all the proteins in addition to conventional analyses.

Lastly, in comparison with typical avian strains, the reversed can be observed for human strains, with almost all showing a signature of 11 exclusive human proteins. There were only 2.19% of the human strains with one avian protein, and the remaining 0.15% with two to eight avian proteins making up the signatures (Fig 2B). In addition, the proportion of human to avian proteins for individual protein predictions were in excess of 99% for all 11 proteins (Fig 2A). Following this finding, this strongly indicates that seasonal influenza viruses circulating in humans have well adapted to human hosts, with efficient transmission between humans.

## Discussion

As evident from the results, the host tropism protein signatures distinguish between avian, human and zoonotic strains based on the number of avian and human viral proteins in the signatures, though there are rare exceptions. Closer observation of the typical avian strain reveals that a very small proportion carries a human NS2 protein (Fig 2A). This could be attributed to prediction error in which the NS2 prediction model does not distinguish between avian or human NS2 proteins as well as the other protein prediction models; or it could also imply that there is less distinction between avian and human NS2 proteins as compared to other influenza proteins. A further investigation found that human NS2 proteins in avian strains are widely distributed across 43 different influenza subtypes in various avian species (S1 Table), which seems to suggest that human NS2 protein can still function well in these avian species. The NS2 or nuclear export protein (NEP), functions to mediate export of viral ribonucleoprotein (vRNP) complexes from host nucleus, as well as control viral RNA accumulation in host cells [60,61]. While it is still unclear if NS2 plays any important role in interspecies transmission of influenza virus, further sequence analysis or molecular studies can perhaps shed light to this interesting observation.

Yet another observation from the results is that proteins from the same segment show vastly different avian-to-human prediction ratios. Contrary to common presumption that proteins from the same segment should be classified similarly, there are higher number of human viral protein predictions of M2, NS1 and PB1-F2 as compared to M1, NS2 and PB1 respectively, despite being coded on the same viral segments. This however, should not be surprising as these pairs of proteins from the same segment (M1 and M2; NS1 and NS2; PB1 and PB1-F2) were not classified purely based on sequence alone, but rather, amino acid physicochemical properties of the protein sequences [10]. As such, even though they originated from the same genomic sequence, they are structurally and functionally different [1,60,62]. Indeed, a study has shown that proteins from the same segment exhibit different evolutionary rates in which both proteins respond individually to structural constraints or selective pressures [63]. Hence, mutations in the gene segment may affect only one protein resulting in a switch in host tropism but not the other. As a result, these protein pairs may play significantly different roles in interspecies transmission, hence maintain a different range of functions in different hosts, ultimately resulting in divergent avian or human classifications. This is due to the individual host tropism classification performed independently for each protein based on the amino acid physicochemical properties observed in the training dataset. Taken together, the host tropism classification of each protein should be considered independently based on the roles they play in avian-to-human transmission of influenza A viruses.

While most zoonotic strains carry host tropism protein signatures with a mosaic of both avian and human viral proteins, there does not appear to be a consistent pattern across all zoonotic strains. Instead, viruses isolated in specific outbreaks show similar signatures. This further highlights the complexity of interspecies transmission, where molecular changes that lead to zoonosis are unique to the outbreak events. Furthermore, although more than half of the zoonotic strains carry between five to ten human viral proteins in the signatures, the signatures of seven zoonotic strains from two separate outbreaks intriguingly contain not a single human protein prediction (S1 Dataset). Such an all-avian signature could be due to prediction error limitations, or they really are avian strains. This is akin to the continuing debate over the entirely avian origin of the deadly 1918 pandemic strain [64–68]. What molecular mechanisms that such avian strains use to overcome the species barrier is yet to be elucidated. One can only speculate that in these rare instances, a non-sequence-based characteristic hitherto not used in existing prediction tools is crucial to the zoonotic process, such as another novel undiscovered viral protein coded in the viral genome, or some host-pathogen interaction too subtle to be determinable from pure sequence analysis and prediction. Nevertheless, the mosaic pattern of host tropism protein signatures can be observed in most zoonotic strains as demonstrated with the zoonotic strains from the 2013 H7N9 outbreak in China and most of the H5N1 zoonotic strains. With increased influenza surveillance and sequencing, additional training samples would increase the accuracy in host tropism classification and hopefully provide more clues in understanding the subtleties in interspecies transmission.

A crucial task following a zoonotic outbreak is to identify the source of infection. The challenge however, is to identify infected avian species which do not exhibit any clinical symptoms, such as one faced by the H7N9 outbreak in China recently [69]. From this analysis, it is evident that we can observe the underlying host tropism protein signatures of avian-isolated suspected zoonotic strains regardless of their clinical symptoms (Fig 2B). With this, we can possibly identify high risk strains in avian species carrying a zoonotic signature of mixed avian and human proteins prior to an outbreak. This could be a useful tool when coupled with adequate influenza surveillance in avian species, providing us with the opportunity for an early alert to an impending species jump into the human population.

Overall, our findings show that the host tropism protein signature is a simplified way of examining the contribution of all proteins in an influenza virus strain. It is by no means comprehensive in itself, nor does it tell with absolute certainty if a strain will be zoonotic given a specific signature. It does however, provide an additional angle from a systemic perspective which could serve to complement traditional analyses including sequence and phylogenetic analyses for more in-depth understanding of the virus. The host tropism protein signatures would therefore be most appropriate for the monitoring of influenza virus strains in the avian population, an epidemiologically useful tool for current influenza surveillance. The capability to identify zoonotic strains that could contribute to the onset of an influenza outbreak may represent a significant advantage over these strains in which we may now possibly identify high risk strains from sequence data alone. If successful, this would allow more time in anticipation of an impending influenza outbreak to reduce public health as well as economic burden.

## Supporting Information

S1 Dataset Complete results of host tropism protein signature analysis of 12,624 influenza A virus strains. (CSV)

S1 Fig Host tropism protein signatures of all complete influenza A virus strain. Each row in the bar represents a strain, with 11 columns depicting one of each protein prediction (HA, M1, M2, NA, NP, NS1, NS2, PA, PB1, PB1-F2, and PB2). Blue column indicates an avian protein prediction while red column indicate a human protein prediction. The upper panel are avian-isolated strains while the lower panel are human-isolated strains. ZA and ZH are avian-isolated suspected zoonotic strains and human-isolated confirmed zoonotic strains from influenza outbreaks.
(PDF)

S2 Fig Host tropism prediction accuracies by protein length. The x-axis represents prediction accuracy of up to 100%, while the y-axis represents the length of the protein. The common protein lengths are represented by the red line [70]. Prediction accuracy decreases for proteins of incomplete length.
(PDF)

S1 Table Distribution of human NS2 proteins across various avian influenza subtypes. (PDF)
