# Supplementary figures and images for: Distinct Host Tropism Protein Signatures to Identify Possible Zoonotic Influenza A Viruses
Source: PLoS One. 2016 Feb 25;11(2):e0150173. doi: 10.1371/journal.pone.0150173 (PMC4767729; doi:10.1371/journal.pone.0150173)

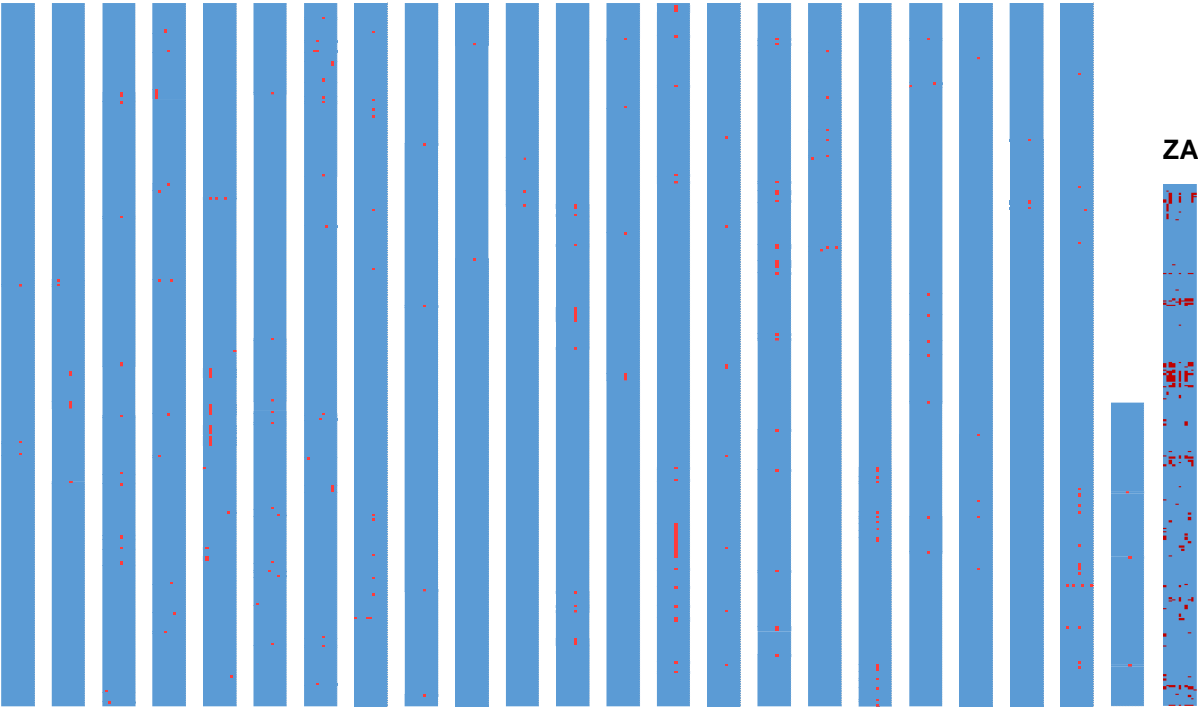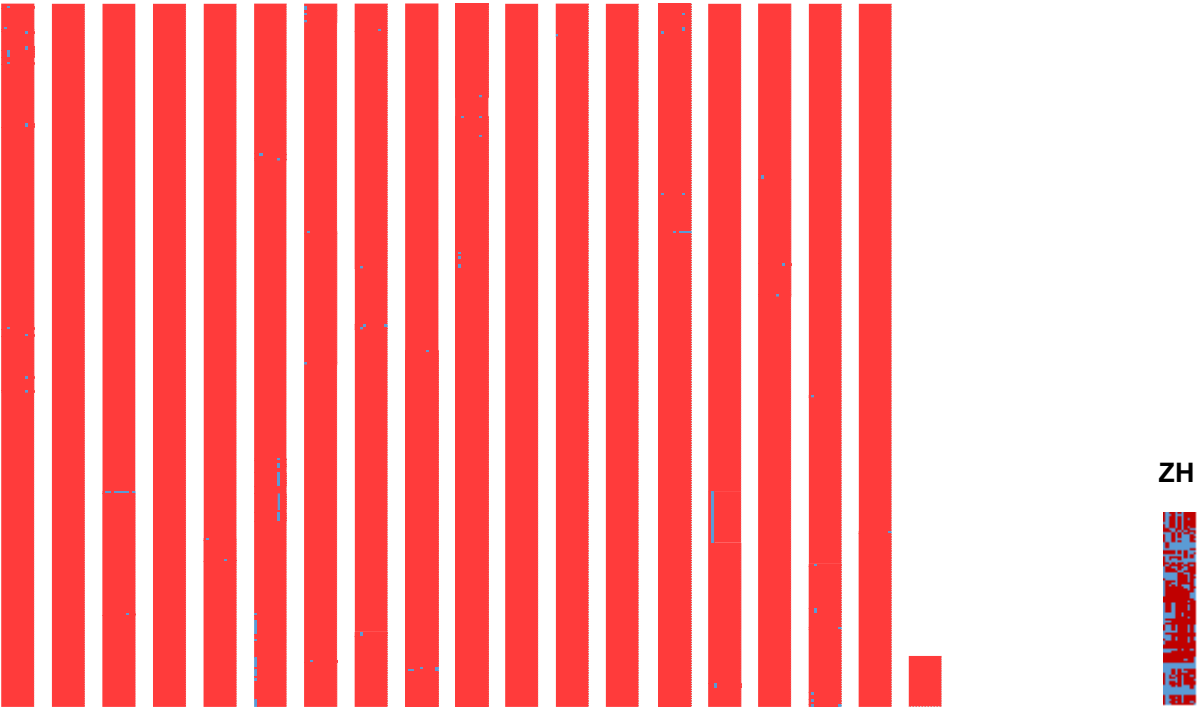

Supplement: S1 Fig — Each row in the bar represents a strain, with 11 columns depicting one of each protein prediction (HA, M1, M2, NA, NP, NS1, NS2, PA, PB1, PB1-F2, and PB2). Blue column indicates an avian protein prediction while red column indicate a human protein prediction. The upper panel are avian-isolated strains while the lower panel are human-isolated strains. ZA and ZH are avian-isolated suspected zoonotic strains and human-isolated confirmed zoonotic strains from influenza outbreaks. (PDF) [file pone.0150173.s002.pdf]

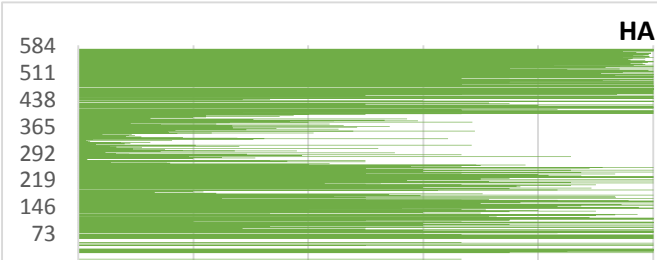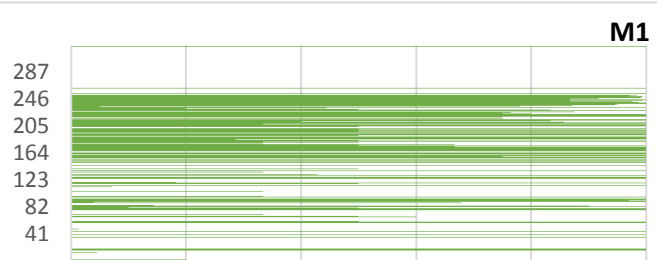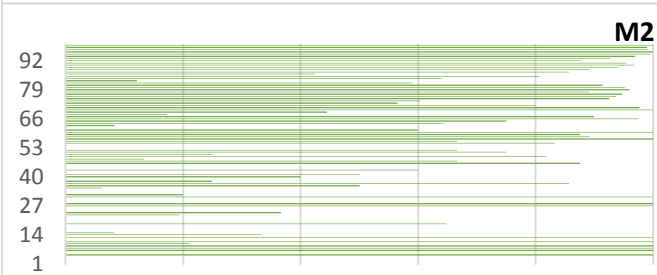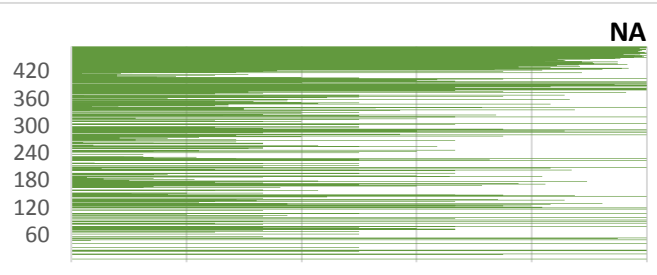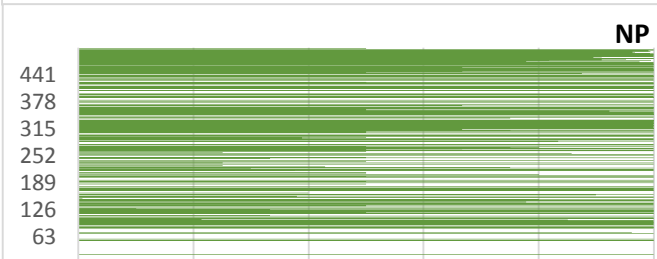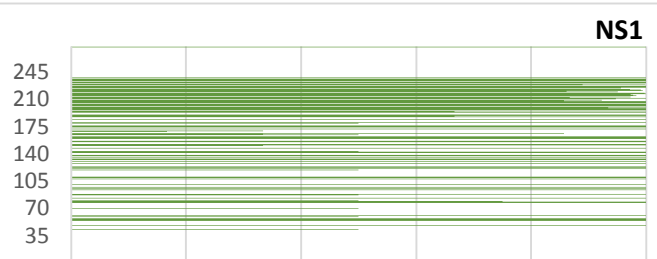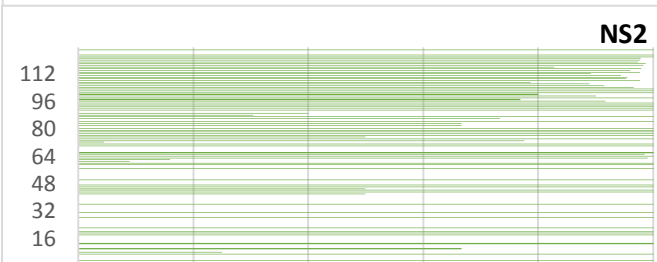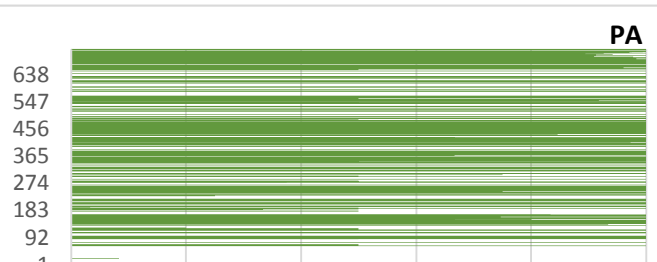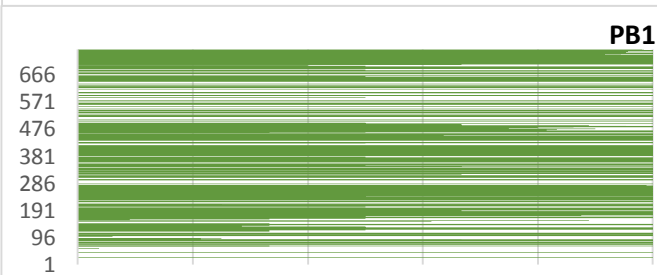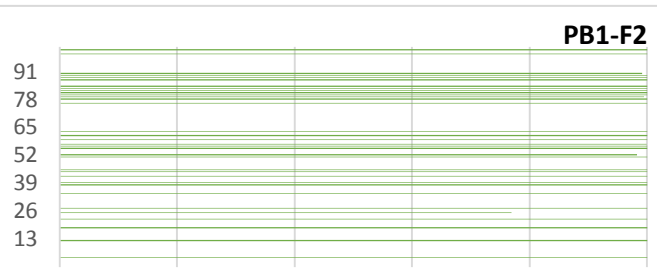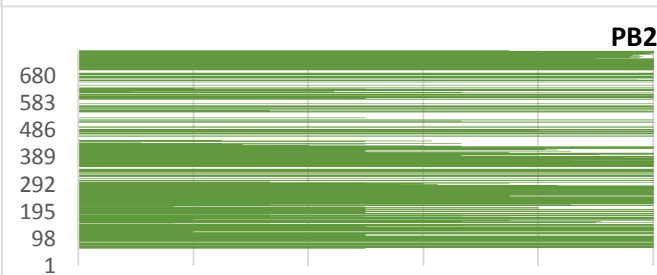

Supplement: S2 Fig — The x-axis represents prediction accuracy of up to 100%, while the y-axis represents the length of the protein. The common protein lengths are represented by the red line [70]. Prediction accuracy decreases for proteins of incomplete length. (PDF) [file pone.0150173.s003.pdf]
