# Supplementary material for: Distinct Host Tropism Protein Signatures to Identify Possible Zoonotic Influenza A Viruses
Source: PLoS One. 2016 Feb 25;11(2):e0150173. doi: 10.1371/journal.pone.0150173 (PMC4767729; doi:10.1371/journal.pone.0150173)
Supplement: S1 Table — (PDF) [file pone.0150173.s004.pdf]

S1 Table. Distribution of human NS2 proteins across various avian influenza subtypes

Subtype	Count
H1N1	12
H1N2	5
H1N6	1
H1N8	1
H1N9	1
H2N1	3
H2N3	4
H2N9	2
H3N2	14
H3N3	1
H3N5	5
H3N6	6
H3N7	1
H3N8	10
H4N2	2
H4N3	1
H4N6	14
H4N8	5
H4N9	1
H5N1	4
H5N2	2
H5N8	1

Subtype	Count
H6N1	3
H6N2	7
H6N5	4
H6N6	1
H6N9	1
H7N3	14
H7N4	2
H7N5	1
H7N6	1
H7N7	25
H8N4	3
H9N2	1
H10N2	1
H10N4	1
H10N7	6
H10N8	2
H10N9	1
H11N2	3
H11N5	1
H11N9	18
H12N5	1
mixed	9
